# Tai Chi Enhances the Effects of Endurance Training in the Rehabilitation of Elderly Patients with Chronic Heart Failure

**DOI:** 10.1155/2011/761958

**Published:** 2010-09-13

**Authors:** Giuseppe Caminiti, Maurizio Volterrani, Giuseppe Marazzi, Anna Cerrito, Rosalba Massaro, Arianna Arisi, Alessio Franchini, Barbara Sposato, Giuseppe Rosano

**Affiliations:** Cardiovascular Research Unit, Department of Medical Sciences, Centre for Clinical and Basic Research, IRCCS San Raffaele, via della Pisana 235, 00163 Roma, Italy

## Abstract

*Purpose*. To assess if Tai Chi added to endurance training (ET) is more effective than ET alone in improving exercise tolerance and quality of life (QOL) of elderly patients with chronic heart failure (CHF). *Design*. Sixty CHF patients, age 73.8 ± 6 years, M/F 51/9, were enlisted. Thirty pts were randomized to combined training (CT) performing Tai Chi +ET and 30 patients to ET (ET only). *Methods*. At baseline and after 12 weeks all patients underwent 6-minute walking test (6MWT), assessment of amino terminal probrain natriuretic peptide (NT-pro BNP), quadriceps maximal voluntary contraction (MVC) and peak torque (PT), QOL questionnaire (MacNewQLMI), blood pressure (BP), and heart rate (HR). All patients performed 4 sessions of exercise/week. *Results*. Distance at 6mwt improved in both groups with significant between-groups differences (*P* = .031). Systolic BP and NT-proBNP decreased significant in the CT group compared to ET (*P* = .025) and *P* = .015), resp.). CT group had a greater significant improvement in physical perception (*P* = .026) and a significant increase of PT compared to ET group. *Conclusions*. The association of Tai Chi and ET improves exercise tolerance and QOL of patients with CHF more efficiently than ET.

## 1. Introduction

Chronic heart failure (CHF) is a disease increasingly recognized as a health burden worldwide. Patients with CHF exhibit impaired exercise tolerance that limits their exercise capacity and quality of life. These symptoms are related to both central and peripheral factors such as structural and functional abnormalities of skeletal muscle [1–3]^.^

It has been widely demonstrated that endurance training (ET) improves aerobic capacity of patients with CHF. Moreover, ET exerts several beneficial effects in CHF patients by improving skeletal muscle structure and function and peripheral blood flow; decreasing neurohormonal function and mortality rate [4–7]. 

Recent studies have evaluated the role of Tai Chi, a kind of low intensity physical activity derived from a traditional Chinese martial art, as a possible nonpharmacological treatment of CHF. Tai Chi seems to improve exercise tolerance, and it could induce favourable hemodynamic changes on CHF subjects compared to usual care [8–10]. Outcome measures of these small studies included quality of life (QOL), exercise capacity, B-type natriuretic peptide, catecholamine levels, blood pressure (BP), arrhythmogenes, and heart rate variability. 

It is not clear if the association of Tai Chi and ET could exert additive effects when administered to CHF subjects already undergoing a cardiac rehabilitation program. Until now no studies have been published on the effects of a combined training (CT) consistent into the association of Tai Chi and ET. 

The first aim of this study was to assess if CT is more effective than ET alone in improving exercise tolerance of elderly patients with CHF. Secondary endpoints were changes in heart rate, amino terminal probrain natriuretic peptide (NT-pro BNP), muscle strength, and QOL.

## 2. Methods

### 2.1. Study Population and Study Design

We enlisted 60 patients with diagnosed CHF due to left ventricular systolic dysfunction, mean age 73 ± 6 years, ejection fraction 33 ± 9%, and M/F 51/9. All patients were in New York Heart Association (NYHA) functional class II. The cause of heart failure was ischaemic heart disease in 48 patients, idiopathic dilated cardiomyopathy in 12 patients. At the time of their enlistment all patients were out-hospital patients, attending to a cardiac rehabilitation program in our day hospital at IRCCS S. Raffaele Istitute of Rome. Inclusion criteria were age >65 years, CHF of more than one year duration due to ischemic or nonischemic dilated cardiomyopathy, left ventricular ejection fraction (LVEF) ≤45%, NYHA functional class II, stable clinical conditions and optimal HF treatment without changes for at least 3 months. Exclusion criteria were history of myocardial infarction or angina less than three months, decompensated heart failure, complex ventricular arrhythmia, angina, and neurological or orthopaedic conditions limiting the exercise protocol. This was an open randomized pilot study. After the completion of baseline testing participants were simply randomized by lot to either the CT group or the ET group. Thirty patients received CT treatment (Tai-Chi+ET) 3 times/week; 30 patients received ET treatment 3 times/week. All patients of the two groups were on top of their medical therapy. 

All patients gave informed consent to participate in the study, which was approved by the local Ethics Committee and conforms to the principles outlined in the Declaration of Helsinki and to the GCP guidelines of the European Community.

### 2.2. Endurance Training

It was performed according to the AHA guidelines [11]. Every exercise session included 10 minutes of warm-up, 10 minutes of cool-down and flexibility exercises, and 30 minutes of aerobic exercise with cycling or walking at 60%–70% of estimated VO2 max.

### 2.3. Tai Chi

In general, Tai Chi is a low-impact, weight-bearing exercise characterized by gentle movements designed to dissipate force throughout the body while the subject changes poses, with well-coordinated sequences of both isometric and isotonic segmental movements in the trunk and 4 extremities. Every session included 10 minutes of warm-up exercises, 30 minutes of Tai Chi practice, and 10 minutes of cooldown exercises. The Tai Chi programme was a modified 10-movement Yang style taught by an experienced Tai Chi instructor similar in style to the routine proposed by Wolf [12]. One-to-two movements were taught each week for 8 weeks. The complete form was practised for the last 4 weeks of the study. Emphasis was placed on performing the movements in a slow, relaxed way. Tai Chi sessions were conducted by an expert Tai Chi trainer with a trained cardiac rehabilitation therapist also in attendance. During Tai Chi practice, subjects imitated the motions and postures of the Tai Chi instructor with the same speed.

In order to balance the total amount of exercise, exercise sessions were organized as follows: all subjects of both groups performed 4 sessions /week. Patients of the CT group performed Tai Chi for 30 minutes two times a week and cycling or walking for 30 minutes 2 times a week (in different days). Patients of the ET group performed cycling or walking for 30 minutes four times a week.

At baseline, in order to estimate their maximal exercise capacity, all subjects performed a symptoms-limited exercise test on a treadmill using a standard Bruce Modified protocol. A resting ECG was performed and repeated at the end of each stage during exercise and during the recovery phases. Data on heart rate and blood pressure were collected during these phases. Maximal oxygen uptake was estimated trough method of heart rate reserve and the training heart rate was estimated based on the following formula. Training heart rate = (maximum HR-resting HR) × desired intensity (%) + resting HR. 

*Changes on exercise tolerance* were evaluated by 6MWT. The test was performed at baseline and at the end of the study according to the standardized procedure [13]. Each test was supervised by a physical therapist. Patients were asked to walk at their own maximal pace a 100 m long hospital corridor. Every minute a standard phrase of encouragement was told. Patients were allowed to stop if signs or symptoms of significant distress occurred (dyspnoea, angina) though they were instructed to resume walking as soon as possible. Results of 6MWT were expressed in distance walked (metres). The Borg scale was used to rate perceived exertion (RPE), and the perceived level of dyspnoea was rated on the Borg category ratio scale [14].

During all the training sessions, HR was assessed in each subject (Polar Team System; Polar Electro Oy), and data were downloaded on a portable personal computer and analyzed using a dedicated software (Polar ProTrainer 5; Polar Electro Oy).

### 2.4. Quality of Life

All patients were administered at baseline and at follow-up examination a vertical visual analog scale to test their quality of life. The visual analog scale was a 10 cm line with a mark at each centimetre. Physical and social QOL were evaluated by MacNewQLMI [15], a self-administered questionnaire previously validated in patients with MI [16, 17], angina [18], and heart failure [19, 20]. MacNew included 27 items in three domains (physical, emotional, and social) with a Global score, has a 2-week duration, and is scored from 1 (low HRQL) to 7 (high HRQL).

### 2.5. Muscle Strength Measurement

Muscle strength measurement: to determine the maximal muscle strength, isometric dynamometry testing (REV9000, Tecno-Gym) of the quadriceps muscles was performed at baseline, and at the end of the study. All measurements were performed while the subject was seated on the device; the chest was fixed by 2 straps, the pelvis and knees flexed at an angle of 90°. The ankle of the tested leg was attached to the strength transducer by a Velcro strip and the patient then carried out 3 consecutive maximal voluntary extensions (contraction time 3 s—resting time 7 s); the highest value was considered as the maximal strength (MVC, N). The isokinetic muscle strength of the knee extensors was assessed by the same dynamometric system, recording the isokinetic strength as torque. Patients performed 5 consecutive knee extension movements with maximal effort and with an angular speed of 90°/s; with the dominant leg the highest value obtained was regarded as the peak torque (PTmax; Nm).

### 2.6. NT-pro BNP Assessment

At baseline and at 12 weeks Venous blood samples were withdrawn from an antecubital vein into chilled ethylene-diamine-tetraacetic acid Vacutainer test tubes after 20 minutes of rest with patients in a supine position. Samples were placed immediately on ice-cold water, and the tubes were then centrifuged at 4000 r.p.m. at 4°C for 15 minutes. NT-pro BNP was determined by a commercially available electrochemiluminescence immunoassay based on a polyclonal antibody-based sandwich chemiluminescence assay (Roche Diagnostics, Germany) using an autoanalyser (Elecsys 2010)

### 2.7. Statistical Analysis

Differences in baseline characteristics between CT and ET groups were evaluated by the chi-square and unpaired *t* test. Within-group changes in the reported variables were evaluated by the paired *t*-test or Wilcoxon signed rank test for nonnormally distributed variables. Between groups comparisons were performed by the unpaired *t*-test and Mann-Whitney rank sum test. The primary and secondary outcomes were evaluated comparing the delta (baseline—12 weeks) of CT versus ET using the Mann-Whitney test. Results are expressed as mean ± SD. A 2-tailed *P* value of <.05 was considered significant. All analyses were performed with a commercially available statistical package (SPSS for Windows version 12.0, Chicago, Illinois).

## 3. Results

Clinical characteristics of the study patients are reported in Table 1. At baseline no differences on anthropometric, clinical, or echocardiographic parameters between the two groups examined were noted. Most of the patients were receiving beta-blockers (88%), angiotensin-converting enzyme inhibitors or angiotensin receptor antagonists (96%), or aldosterone receptor blockers (63%); 58% were taking diuretics, and 36% were receiving digitalis. Medications were not altered throughout the study.

Patients of CT group trained at a variable intensity (during endurance training: at 66 ± 5% VO2; during Tai Chi practice at 52 ± 3% VO2). Conversely patients of ET group had a constant intensity of training (at 67 ± 4% VO2).

### 3.1. Exercise Tolerance and Hemodynamic Profile

After 12-weeks of treatment, NYHA functional class improved in 36% of CT and 32% of ET without between-groups differences. Distance walked at 6 MWT increased (from 214 ± 32 m to 291 ± 46 m; *P* = .0001) in the CT group and in the ET group (from 219 ± 23 m to 272 ± 33 m; *P* = .0001) with significant between-groups difference (*P* = .031) (Table 2). The Borg scale decreased in both group (CT −2.1 ± 0.3; ET 1.9 ± 0.4; resp.) without significant between-groups differences (*P* = .24). 

Rest HR decreased by 11.2% beats/min in the CT group and by 6.8% beats/min in the ET group (between groups *P* = .074). Systolic BP decreased by 12.3% in the CT group and by 6.0% in the ET group with significant between-groups differences (*P* = .025). Diastolic BP decreased in both groups at a similar degree. NT-proBNP levels decreased in both groups (CT = −28%; ET = −18%) with significant between-groups differences (*P* = .015). 

PT of the quadriceps had a higher significant increase in the CT group compared to ET (*P* = .037) (Table 2). There was a trend toward a greater increase of MVC in the CT groups compared to ET (*P* = .069). 

### 3.2. Quality of Life

After 12-weeks, the overall assessment of QOL by a visual analog scale showed a similar improvement in patients of groups compared to baseline (CT: from 4.1 ± 0.6 to 6.4 ± 0.8); (ET: from 4.3 ± 0.7 to 6.2 ± 0.9) without significant between-groups differences (*P* = .137). Physical QOL evaluated by MacNewQLMI improved in patients in the CT group to a greater extent than ET group (+32.3% versus +23%, between groups *P* = .026). Patients of the CT group had a trend to a greater improvement on social (+12% versus +5%; *P* = .058) and emotional (+9% versus +6%; *P* = .083) QOL than ET group.

### 3.3. Clinical Outcome

Tai Chi and ET were both well tolerated. No patients had adverse events during the exercise protocol. No patient died during the follow-up period. Worsening HF occurred in 1 patient of the ET group and was managed only by increasing the dose of furosemide. None patient required hospital stay during the study period. Three patients (10%) of the ET group withdraw from the study.

## 4. Discussion

The present pilot study shows two original results. First, we demonstrated that a CT including Tai Chi and ET improves exercise tolerance and quality of life of elderly patients with CHF to a greater extent than ET alone. Second, our data suggest that CT could lead to a better hemodynamic profile compared to ET alone. 

We observed a 36% increase of distance walked at 6MWT in the CT group. This improvement of exercise capacity resulted significantly higher compared to the group performing only ET (24% increase) and appears considerably better than those reported by other studies where the increase of functional capacity after Tai Chi was ranging from 7% to 26% [8–10]. However, some significant differences between ours and other studies should be underlined. At first we enlisted only patients in stable clinical conditions at early-intermediate stage of the disease (only NYHA class II). Conversely, in the study of Barrow et al. [10] an unspecified number of patients enrolled were in the NYHA class III. Moreover, for the first time we evaluated the combined effects of ET associated to Tai Chi on exercise capacity. In fact previous authors have suggested Tai chi only as a safe alternative to conventional low intensity exercise training, particularly for subjects with CHF who are not suitable for ET because they are elderly or in very advanced stage of the disease. Conversely, our results suggest a new role for Tai Chi in the contest of the cardiac rehabilitation program of CHF subjects who are contemporaneously performing ET. The mechanisms by which CT enhances the effects of ET are unclear. Our data suggest that they could be related to a combination of physical and psychological factors. Among physical factors we would distinguish central and peripheral variables. 

### 4.1. Central Variables

In our study a 12-weeks CT lead to a significant 12.3% reduction of systolic BP compared to 6.4% of ET alone. Our data confirm previous observations: a reduction of mean blood pressure has been found for regular Tai Chi practice in several studies. A recent review of twenty-six studies showed that twenty-two of them reported reductions in BP with tai chi (3–32 mm Hg systolic and 2–18 mm Hg diastolic BP reductions [21]). Tai Chi seem to have similar effects ET on systolic BP. Young et al. [22] found changes in systolic BP, after 12 week of −8.4 mm Hg and −7.0 mm Hg in the aerobic exercise and Tai Chi groups, respectively. The effects of CT on HR and BP could be the result of a stronger modulation of neurohormonal activity in the CT group activity as demonstrated by other authors [23]. We observed a significant reduction of NT-proBNP levels in the CT group compared to ET. A similar reduction of natriuretic peptide was registered by Yeh et al. [9] who compared Tai Chi versus usual care in patients with CHF. We speculate that the reduction of NT-proBNP levels could be related to an improvement of cardiac filling pressure in subjects performing CT to a greater extent than ET.

### 4.2. Peripheral Variables

We observed a significant increase of isokinetic strength of quadriceps in the CT group compared to ET. Isometric strength presented also a trend toward a greater increase in the CT group than ET. Objective morphological and functional abnormalities, relatively independent of reduced blood flow, are present in the muscle of CHF patients [24]. These maladaptive changes in the muscles, which include fiber atrophy and a prevalence of type II fibers, are involved in symptoms development. Moreover, low muscle strength is related to poor outcome in CHF patients [25]. Exercise training is effective in improving muscle strength in elderly people [26] and patients with heart failure [27]. Significant improvement in muscle strength in knee and ankle flexors and extensors have been demonstrated in elderly healthy people after Tai Chi [28, 29] while, to our knowledge, no studies have evaluated the effects of Tai Chi on muscle strength in patients with CHF. Our data suggest that Tai Chi could enhance the effects of ET also in peripheral factors.

According to our data CT training seems to be superior to ET alone also in the psychological and motivational field. In our study patients allocated to CT group had a significant increase of physical perception compared to ET. Our data are in line with other studies who evaluated the effects of Tai Chi on psychological responses in subjects with cardiovascular diseases [30–32]. In these studies Tai Chi have shown improvement compared with the control group in several indices of psychological well-being targets such as depression, psychological distress, positive well-being, life satisfaction, and perceptions of health. In the recent randomized pilot study of Barrow et al. [10] on patients with symptomatic CHF, a 16-week Tai Chi program leads to a significant improvement of depression scale compared to usual care.

### 4.3. Study Limitations

Our study included only 9 (15%) female patients and we can not generalize our results to female gender. We made a pilot study with a small sample size and our data should be confirmed in further well-powered studies. Despite our data on blood pressure and HR suggest strong effects of CT on autonomic nervous system we did not directly measure the neurohormonal activity in our patients. 

In conclusion, our study suggests that the association of Tai Chi to ET can improve exercise tolerance of patients with CHF to a greater extent than ET alone. Moreover, CT seems to determine better hemodynamic and psychological effects than ET. CT could play an important role in the rehabilitation of patients with chronic heart failure in stable clinical conditions.

Results of this pilot study are promising, considering its short duration and the large magnitude of improvement in cardiovascular fitness observed.

## Figures and Tables

**Table 1:** Baseline Features of the Overall Population and Patients of CT and ET groups.

	Overall population	CT (*N* = 30)	ET (*N* = 30)
Age, years	73.8 ± 6	74.1 ± 6	73.4 ± 2
M/F	51/9	25/5	26/4
Cause of heart failure			
Ischemic heart disease	47	24	23
Idiopathic dilated cardiomyopathy	13	6	7
BMI	27.6 ± 3	27.8 ± 2	27.2 ± 3
Hypertension	42	19	23
Atrial fibrillation	12	7	5
Diabetes	28	15	13
COPD	23	11	12
Echocardiography			
LVDD, mm	60.1 ± 5	59 ± 4	60 ± 6
LVSD, mm	45.9 ± 4	45 ± 5	46 ± 5
Ejection fraction, %	33.0 ± 9	33.6 ± 9	32.8 ± 12
Treatment			
Beta-blockers	53	26	27
ACE-i/ARBs	58	28	30
Diuretics	35	18	17
Aldosteron-antagonists	38	18	20
Statins	42	23	19
Digitalis	22	13	9

BMI: body mass index

LVDD: left ventricular diastolic diameter

LVDD: left ventricular systolic diameter.

**Table 2:** Comparison of Delta (Baseline versus 12 Weeks) of Hemodynamic, and Muscle Strength in the CT and ET Groups. Data Are Expressed as Mean ± Standard Deviation.

	CT group (*n* = 30)			ET group (*n* = 30)			Between-groups comparison
	Baseline	12 weeks	Delta	Baseline	12 weeks	Delta	(*P*)
Exercise tolerance							
6MWT, m	214.9 ± 32	291.5 ± 46	77.4 ± 26	219.2 ± 23	272.0 ± 33	53.2 ± 16	.031
Hemodynamic							
Heart rate, bpm	72.3 ± 12	64.5 ± 11	−8.2 ± 2.2	74.6 ± 11	69.5 ± 9	−5.7 ± 1.6	.074
Systolic BP, mmHg	131.3 ± 26	115.6 ± 23	−13.4 ± 2.4	134.1 ± 29	127.7 ± 31	−6.4 ± 1.3	.025
Diastolic BP, mmHg	84.3 ± 14	79.6 ± 13	−5.6 ± 1.4	83.9 ± 17	79.6 ± 14	−4.3 ± 0.7	.66
NT pro-BNP, pg/mL	136.4 ± 31	99.7 ± 22	−36.7 ± 8	134.5 ± 28	111.7 ± 24	−22.8 ± 7	.015
Muscle strength							
MVC, N	65.4 ± 15	83.6 ± 19	18.2 ± 4	66.0 ± 11	80.1 ± 20	14.1 ± 3	.069
PT, Nm	41.3 ± 17.4	68.2 ± 9	26.9 ± 7	42.6 ± 17.4	56.7 ± 11	19 ± 1	.037

6MWT: 6 minutes walking test

BP: blood pressure

NT pro-BNP: amino terminal probrain natriuretic peptide

MVC: maximal voluntary contraction

PT: peak torque.
